# Dental arch morphology and factors associated with dental crowding: a systematic review

**DOI:** 10.3389/froh.2026.1784622

**Published:** 2026-06-08

**Authors:** Ana Isabel Contreras-Madrid, David Pérez-Jorge, Roshan Melwani-Sadhwani, Susell Parra-Rojas, Rocío Trinidad Velázquez-Cayón, Juliana Cassol-Spanemberg

**Affiliations:** 1 Doctoral Program in Health Sciences, University of La Laguna, San Cristóbal de La Laguna, Spain; 2 Department of Didactic and Educational Research, University of La Laguna, San Cristóbal de La Laguna, Spain; 3 Department of Dentistry, Faculty of Health Sciences, Fernando Pessoa Canarias University, Santa María de Guía de Gran Canaria, Spain

**Keywords:** dental arch, dental crowding, etiology, gender, malocclusion

## Abstract

**Introduction:**

Dental crowding is considered the most common type of malocclusion, ranking third among the most prevalent oral pathologies. Several genetic and environmental factors are associated with dental crowding. Dental arch dimensions are essential for determining the degree of crowding in the dentition. This review aims to assess the influence of dental arch dimensions on the prevalence of crowding.

**Material and methods:**

A study was conducted in accordance with PRISMA guidelines and registered in PROSPERO (CRD420251082613). An electronic literature search from 2010 to 2025 was conducted, selecting articles in English and Spanish. PubMed, Web of Science, and Scopus were used as data sources; all keywords were included as MeSH terms.

**Results:**

Based on 14 articles, 10 cross-sectional and 4 longitudinal studies, of medium and high quality according to the risk of bias analysis.

**Conclusions:**

Arch dimensions are associated with the occurrence and severity of dental crowding. There is no unanimity in the results on whether it is more frequent in males or females. The combination of larger tooth size and reduced arch dimensions is consistently associated with dental crowding. With increasing age, arch length tends to decrease, often associated with anteroinferior crowding. However, the relative contribution of these factors remains insufficiently quantified, highlighting the need for longitudinal studies with standardized methodologies. These findings may support a more comprehensive and individualized approach to the assessment and management of dental crowding.

**Clinical significance:**

Understanding the relationship between arch dimensions and crowding is essential for orthodontists. It supports diagnostic decision-making based on individualized morphometric assessment and may improve early identification of patients at risk of developing crowding.

## Introduction

Dental crowding is the most common malocclusion and is characterized by mesiodistal overlapping of adjacent teeth within the same arch due to a negative discrepancy between the space required to accommodate the teeth and the space available in the dental arch (1–5). From a three-dimensional perspective, crowding represents a complex disharmony between tooth size, arch perimeter, and skeletal growth patterns. This discrepancy can cause rotations, misplacements, and tooth overlap, even in the absence of other evident dental anomalies.

Although dental crowding is not considered a disease, it is associated with the onset of several pathologies, particularly periodontal disease and dental caries (5). The accumulation of plaque in malaligned teeth complicates hygiene and increases the risk of gingival inflammation, clinical attachment loss, and localized demineralization in both adolescents and adults.

Several studies (6–10) agree that both genetic and environmental factors contribute to the etiology of dental crowding. Cordoví et al. (6) highlight the relevance of inflammatory responses in oral tissues and their relationship with immune and structural conditions. Amaral and López (9), in a study conducted in Tabasco, Mexico, report a high prevalence (83.7%) of late mandibular crowding, with over 50% of cases classified as severe or very severe. These findings underline the importance of dentoalveolar discrepancies and skeletal growth patterns in the development of crowding.

Amaral and López (9) and Delgado Sánchez (10) identify various factors associated with crowding, including the position of permanent tooth germs, the timing of deciduous tooth exfoliation, tooth size, mandibular development, and condylar growth. Specifically, Amaral et al. found that 26% of cases showed reduced mandibular length and that crowding occurred independently of the presence or position of third molars. These observations suggest that arch morphology plays a more significant role than previously attributed to specific dental elements, such as third molars.

The dimensions of the dental arch are essential in determining the degree of crowding. Smaller arches and larger teeth are consistently associated with crowding (11). Tooth size must be matched to the bony base to avoid space discrepancies. In the Tabasco study, 70% of the subjects with crowding had dentoalveolar discrepancies in the lower arch, reinforcing the idea that mismatches between arch length and tooth size are key contributors to the condition. Furthermore, although both crowded and non-crowded individuals showed predominantly horizontal mandibular growth, none of the individuals with vertical growth patterns exhibited late mandibular crowding (9).

**Figure 1:**
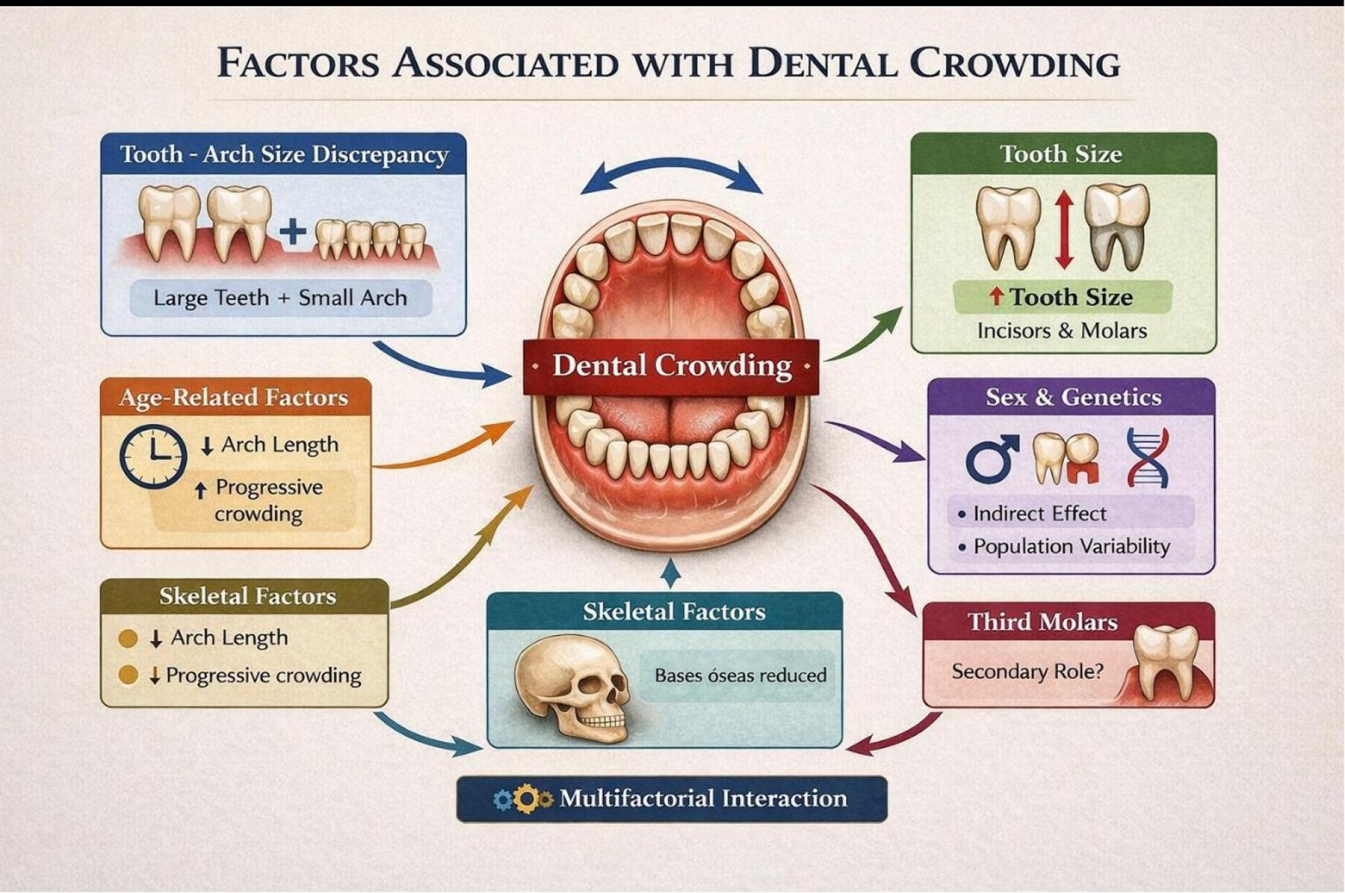
Multifactorial model of dental crowding development.

In orthodontics, the role of third molars in contributing to crowding, particularly in the anterior mandibular segment, remains controversial. It has been hypothesized that erupting third molars can transmit mesial forces through the dental arch, leading to anterior tooth displacement. Khalid et al. (2) argue that this force may result in reduced anterior space and crowding, although most studies reviewed by the authors do not support a causal relationship. Their findings suggest that any association may be coincidental, given that third molars erupt during a period when other growth-related changes are occurring. This interpretation aligns with the conclusions of (2, 3, 12), all of whom highlight the multifactorial nature of anterior crowding and the limited role of third molars in its development. As illustrated in Figure 1, dental crowding is influenced by multiple interacting factors, including tooth size, arch dimensions, skeletal patterns, and genetic and environmental components.

Therefore, although third molars have been historically implicated in late crowding, current evidence suggests their role may be overestimated. More consistent factors across the literature include arch length, tooth size, skeletal growth patterns, and sexual dimorphism (9, 11–13).

Regarding sexual dimorphism, several studies have reported that sex-related differences in arch dimensions and tooth size may influence the prevalence and severity of crowding. Stanaitytė et al. (12) report that crowding is more common in females, who tend to have narrower and shorter arches, while males often have greater arch length and width, offering more space for alignment. While some biometric studies have identified a stronger influence of mesiodistal tooth width in males, others, like (9), found no statistically significant difference in crowding prevalence by sex, suggesting that the morphological traits associated with each sex may indirectly influence the development of crowding, rather than act as direct determinants.

Given the heterogeneity of findings in the scientific literature regarding the relationship between dental arch dimensions and crowding, a systematic review of the articles published to date has been undertaken. The general aim of this review is to analyse the influence of dental arch dimensions on crowding. The specific objectives are: (a) to examine how arch dimensions affect the presence and severity of dental crowding; (b) to determine whether tooth size influences its occurrence; and (c) to assess whether a reduction in arch length over time contributes to the development of dental crowding. Additionally, this review explores whether the reviewed studies report differences in these factors based on sex.

### Material and methods

This systematic review was conducted in accordance with the PRISMA (Preferred Reporting Items for Systematic Reviews and Meta-Analyses) (38) guidelines to ensure transparency and objectivity in the identification, selection, and analysis of the included studies (16). The study was registered in PROSPERO (CRD420251082613) and conducted based on the PECO framework. To guide the search, the following research question was formulated: *Do dental arch dimensions influence the occurrence of dental crowding?*

According to the PECO framework, the identified keywords were: dental crowding, malocclusion, etiology, and dental arch. A literature search was conducted covering the period from 2010 to 2025, selecting articles in English and Spanish from studies conducted in humans. The data sources used were PubMed, Web of Science, and Scopus, and all keywords were included as MeSH terms. The search strings used were as follows:

**WOS:** (“dental crowding” AND (malocclusion OR “dental arch”) AND etiology)
**Scopus:** (“dental crowding” AND “etiology” AND (malocclusion OR “dental arch”))
**PubMed:** (“dental crowding” [MeSH Terms] OR “dental crowding”) AND (“malocclusion” [MeSH Terms] OR malocclusion) AND (“dental arch” [MeSH Terms] OR “dental arch”) AND (etiology OR causes OR risk factors) (dental crowding [MeSH Terms]) AND (dental arch [MeSH Terms])

Selection criteria: Inclusion criteria were cross-sectional, case-control, and cohort studies involving participants without prior or ongoing orthodontic treatment and addressed dental crowding specifically, excluding other malocclusion types. Only manuscripts written in English or Spanish with rigorous methodological designs—quantitative, qualitative, or mixed—were considered. Eligible studies involved participants without genetic syndromes or systemic conditions linked to malocclusions. Excluded were studies on cadavers, animal models, or computational simulations, as well as narrative reviews, expert opinions, letters to the editor, single case reports, and clinical studies lacking systematic analysis or quantifiable data.

Grey literature was explored through manual searches on platforms such as Google Scholar (first 100 titles) and OpenGrey. Additionally, reference lists of selected articles were manually reviewed for further relevant studies. The final search was completed on May/25. The methodological quality of the included studies was assessed through the selection of research employing rigorous methodological designs, emphasizing transparency, credibility, and relevance in addressing the research objectives.

The Joanna Briggs Institute (JBI) critical appraisal tool was used to assess the risk of bias of the included studies. Each study was independently evaluated across relevant methodological domains according to its design (e.g., cross-sectional or cohort). The overall quality of the studies was categorized as moderate or high based on the number of criteria met and the consistency of the methodological rigor reported. Any discrepancies between reviewers were resolved through discussion and consensus. The results of the quality assessment were summarized in Table 1.

**Table 1:** Summary of zincluded studies: main findings by sex, arch size, tooth size, and arch length/apical base.

Year & Author	Sample	Sex	Arch Size	Tooth Size	Arch Length/Apical Base
Travassos da Rosa et al. (14)	*n* = 40; 24 females and 16 males	No sex differences	In the lower arch, increased crowding was inversely associated with tooth wear and changes (decreases) in arch perimeter.	Dental wear: Increase between 0.65 and 0.99 units. Anterior crowding was measured using the Little Crowding Index.	Slight increase (<1 mm) in anterior dental crowding and decrease in arch perimeter (<1.5 mm).
Singh and Shivaprakash (15)	*n* = 60; Group I ≥ 3 mm crowding; Group II <3 mm	NA	NA	No difference between groups	Shorter mandibular length in ≥3 mm crowding; inverse correlation with crowding
García-Gil et al. (16)	*n* = 100; 46 females and 54 males	A comparison of sex revealed that males showed specific larger spaces, arch lengths, tooth sizes, lower posterior arches, and upper and lower intermolar widths, as well as a steeper mandibular plane when compared to females.	*Maxillary arch length*: mean 36.74 mm *Mandibular arch length*: mean 26.09 mm	*Maxillary posterior tooth size*: mean 45.66 mm *Mandibular anterior tooth size*: mean 23.21 mm	Total upper arch length (male's mean: 40.62 mm; vs. female's mean: 38.73; *p* = 0.001) and anterior upper arch length (male's mean: 14.86 mm; vs. female's mean: 13.25; *p* = 0.001) were larger in males than females. Posterior lower arch length (male's mean: 27.33 mm; SD: 1.72 vs. female's mean: 26.08; *p* = 0.05) was larger in males too.
Singh et al. (17)	*n* = 152; 4 groups by crowding/spacing	NA	NA	NA	≥3 mm crowding group: smaller mandibular base; weak-moderate inverse correlation with base length
Paul et al. (18)	*n* = 60; Normal <1.6 mm; Crowded >1.6 mm	More crowding in females (NS); females smaller intermolar width	Smaller intermolar width in females	Total incisor width higher in crowded group	Shorter arch length in crowded group
Das et al. (19)	*n* = 132; 33 men/women with & without crowding	In men, larger MD crown width & smaller arch linked to crowding; not in women	Smaller arch perimeter in crowded group	Larger tooth size in crowded group	NA
Montasser and Taha (20)	*n* = 45; Group 1 < 3 mm; Group 2 ≥ 3 mm	NA	NA	NA	Strong correlation between maxillary & mandibular base lengths; no correlation with crowding
Saito et al. (21)	*n* = 12; Control (10), Case (12)	NA	Decreased anterior arch width & lingual canine inclination increased crowding	NA	Age-related increase in maxillary crowding due to reduced arch length
Rojas-Sánchez et al. (22)	*n* = 63	No sex differences	Significant lower interpremolar distance in severe crowding	No significant differences, higher in men	No significant differences
Daoud et al. (23)	*n* = 100	No sex differences	Maxillary arches more crowded	Crowded arches: higher crown ratios (upper incisors, lower lateral incisors)	Negative correlation: tooth diameter & arch length
Normando et al. (24)	*n* = 107 (4 ethnic groups)	No sex differences	Larger maxillary arch in Assurini	Larger maxillary teeth in Assurini	Shorter mandibular arch (2.5 mm) in Arara-Laranjal
Crossley et al. (25)	*n* = 75	Females: smaller tooth size & arch; Males: larger apical base	Mandibular size unrelated to crowding; smaller maxillary base linked to more crowding	NA	Mandibular length related to arch perimeter, intermolar & intercanine widths
Milos et al. (26)	*n* = 61	More jaw growth in males	Decreased arch depth	NA	NA
Mauad et al. (27)	*n* = 20	NA	Significant intermolar width decrease (0.43 mm)	NA	Arch length reduced: right side 1.28 mm → 0.51 mm; left side 0.77 mm

NA, not acknowledged.

## Results

This systematic review synthesized findings from 14 studies, 10 cross-sectional and 4 longitudinal, classified as moderate to high quality according to the JBI risk-of-bias assessment. The studies evaluated the relationship between dental crowding and four main variables: sex, arch size, tooth size, and arch length. The results are presented below by theme and in the Table 1. The included studies showed considerable methodological and demographic variability, including differences in dentition stage, population background, and measurement protocols, in participant characteristics such as age ranges (from mixed dentition to adulthood) and population backgrounds (e.g., Indigenous, Afro-descendant, and Asian populations), although this information was inconsistently reported across studies. Of the included studies, four were longitudinal, providing higher-level evidence regarding temporal changes in dental arch dimensions and the progression of dental crowding, particularly in relation to age-related effects.

### Sex and dental crowding

A few studies analyzed the association between sex and dental crowding. Paul et al. (18) and Crossley et al. (25) observed a trend of greater crowding in females, potentially due to reduced intermolar width; however, these findings were not statistically significant. Das et al. (19) found that increased mesiodistal crown width in males correlated with crowding, while no such association was found in females.

### Arch size

Most studies identified a relationship between smaller arch size and increased dental crowding. Paul et al. (18), Rojas-Sánchez et al. (22), and Das et al. (19) reported smaller arch perimeters in individuals with crowding. Saito et al. (21) observed a correlation between decreased anterior arch width and crowding, including mandibular canine inclination. Mauad et al. (27), in a longitudinal study, recorded a reduction of 0.43 mm in intermolar width over 11 years. Normando et al. (24) found morphological differences between Indigenous populations in Brazil, suggesting a genetic influence on arch dimensions and crowding.

### Tooth size

Larger tooth size was associated with crowding in several studies. Paul et al. (18) and Das et al. (19) reported a statistically significant increase in anterior tooth width among individuals with crowding. Daoud et al. (23) found increased size of maxillary central and mandibular lateral incisors, as well as larger molars, in crowded cases. García-Gil et al. (16) associated crowding with specific posterior eruption sequences and larger posterior teeth in the maxilla and both anterior and posterior teeth in the mandible. However, Singh and Shivaprakash (15) did not observe a significant correlation when comparing groups by crowding severity, and (17) suggested that tooth size alone is not a decisive factor.

### Arch length

Arch length showed the most consistent inverse relationship with dental crowding across the studies reviewed. Reduced mandibular arch length was significantly associated with crowding in several cross-sectional studies (15, 17, 18, 21).

Importantly, the longitudinal studies included in this review provide stronger evidence supporting age-related changes. Mauad et al. (27) observed a progressive reduction in arch length over time, accompanied by an increase in crowding. Similarly, Travassos et al. (14) reported increasing dental crowding and arch reduction over a 13-year period, suggesting that these changes may be influenced by factors such as reduced interproximal wear and masticatory function.

## Discussion

This manuscript explores the relationship between dental crowding and four primary variables: sex, arch size, tooth size, and arch length. Overall, the findings reveal consistent trends regarding arch dimensions and tooth size, whereas results related to sex and growth patterns remain heterogeneous. Taken together, the evidence supports a multifactorial and predominantly associative interpretation of dental crowding rather than a static or unidimensional phenomenon.

An important source of heterogeneity across the included studies is related to demographic variability. Differences in age distribution and population background may influence dental arch development and crowding patterns, particularly through age-related processes such as late mandibular growth and mesial drift, as well as genetically determined variations in craniofacial morphology across populations. However, the inconsistent reporting of these variables across studies limits the ability to perform subgroup analyses and may partially explain the variability in findings. This heterogeneity is further influenced by differences in dentition stage, measurement protocols, and study design, which limit direct comparability across studies and preclude quantitative synthesis.

These factors effectively act as implicit subgrouping variables that contribute to the variability of findings across studies.

Although sexual dimorphism in dental arch morphology has been widely described, its direct role in the development of dental crowding remains inconclusive. Paul et al. (18) and Crossley et al. (25) identified a tendency toward greater crowding in females, attributed to narrower arch widths, particularly intermolar width. These findings are consistent with previous studies indicating that females generally present shorter and narrower dental arches (12). In contrast, Das et al. (19) found that increased mesiodistal crown width in males correlated with increased crowding, highlighting that tooth size may be a more critical factor than arch size in male subjects.

Population-based studies further highlight the lack of consensus regarding sex differences in crowding prevalence. Vaishali et al. (28) reported a higher prevalence of crowding in males, while (22, 29) found no statistically significant differences by sex. Amaral and López (9) also found no significant sex-related difference in late mandibular crowding. These inconsistencies may reflect differences in age distribution, genetic background, growth patterns, and methodological heterogeneity across studies. Collectively, these findings suggest that sex-related differences influence dental crowding indirectly, through variations in arch morphology and tooth size, rather than acting as an independent etiological factor.

Arch dimensions consistently emerge as a key factor associated with dental crowding, supporting the classic theory of (1), which posits that spatial discrepancies between arch length and tooth size are central to malalignment. Several studies in this review, including those by (18, 19, 22), confirmed that individuals with crowding exhibit smaller arch perimeters and reduced intercanine or intermolar widths. These findings are consistent with earlier biometric analyses by (11, 13). Taken together, these data reinforce the central role of arch morphology in determining space availability within the dental arches.

Longitudinal data further reveal that arch constriction can progress over time. Mauad et al. (27) documented a 0.43 mm reduction in intermolar width over 11 years (21) reported progressive anterior crowding in adults, linked to decreased arch width and changes in incisor inclination. Sardarian and Ghaderi (30) confirmed an inverse relationship between intercanine width and crowding in mixed dentition. Importantly, longitudinal studies provide stronger evidence than cross-sectional analyses, as they consistently demonstrate progressive arch length reduction accompanied by increasing anterior crowding with age, underscoring the dynamic nature of this condition.

These findings further reinforce the role of age-related changes in the development and progression of dental crowding. Normando et al. (24), in their study of Amazonian Indigenous populations, found marked variations in arch morphology and tooth size across tribes, suggesting that both genetic and environmental factors shape arch development and the risk of crowding. These findings emphasize that arch dimensions must be interpreted within a broader biological and population-specific context.

The correlation between tooth size and crowding is well documented. Studies included in this review, such as (18, 19), confirmed that increased mesiodistal crown width, particularly of incisors and molars, is associated with reduced arch space. This finding supports the conclusions of classic biometric studies (11, 31, 32), which linked larger tooth dimensions to space deficiencies. Daoud et al. (23) observed that both anterior and posterior teeth contribute cumulatively to crowding, especially when combined with reduced arch dimensions. García-Gil et al. (16) added a novel perspective by identifying specific eruption sequences that predispose to crowding.

Nevertheless, Singh and Shivaprakash (15) and Singh et al. (17) argued that while tooth size is a contributing factor, it alone is insufficient to explain crowding. These observations support the notion that the interaction between tooth size and arch morphology, commonly referred to as the tooth-size/arch-length discrepancy, determines dental crowding.

Arch length showed the strongest and most consistent association with dental crowding across all variables. Reduced mandibular arch length was significantly associated with crowding in the studies of (15, 18, 21). Mauad et al. (27) provided longitudinal evidence of arch length reduction with age, paralleling a measurable increase in anterior crowding. These findings further support the view of crowding as an age-related, progressive phenomenon.

Skeletal base dimensions also play a role, as evidenced by (33), who found shorter maxillary and mandibular base lengths in Class II patients with moderate to severe crowding. Montasser and Taha (20) and Türkkahraman and Sayin (34) further emphasized that dentofacial morphology may mediate space availability and crowding patterns, findings that are consistent with studies evaluating dentoskeletal effects and arch dimension changes following orthodontic treatment (39, 40). Accordingly, skeletal structures appear to act as modulators of crowding rather than as isolated causal factors. In addition, clinical case reports have illustrated that severe dental crowding may be associated with complex skeletal and dentoalveolar discrepancies requiring multidisciplinary management, highlighting the potential clinical relevance of morphometric findings in extreme presentations (35, 36).

Historical and anthropological data also provide insights. Herrera-Atoche et al. (37) analyzed a 13,000-year-old skull, noting reduced masticatory forces and modern-like arch dimensions as contributors to malocclusion and crowding. Travassos et al. (14) observed similar trends over 13 years in Amazonian populations. These observations reinforce the concept of dental crowding as a dynamic condition shaped by evolutionary, functional, and age-related factors.

Beyond summarizing existing evidence, this review provides a structured synthesis of the multifactorial nature of dental crowding by integrating tooth size, arch morphology, skeletal base characteristics, and age-related changes within a unified framework. Importantly, it highlights key inconsistencies in the literature, particularly regarding sexual dimorphism, and identifies major sources of heterogeneity, including demographic variability and differences in study design. The limited availability of longitudinal studies and the inconsistent reporting of population characteristics further underscore the need for more standardized and high-quality research in this field. The variability in methodologies, population characteristics, and outcome definitions among the included studies prevented the calculation of standardized effect estimates and limited the feasibility of formal subgroup analyses.

Taken together, the findings of this systematic review suggest that dental crowding is associated with a complex and multifactorial interaction between tooth size, arch morphology, skeletal base characteristics, and age-related changes; however, these associations should be interpreted with caution given that most included studies are cross-sectional in design.

While sexual dimorphism influences arch dimensions and tooth size, its role appears to be indirect and context-dependent rather than etiological.

### Limitations

In this review, only tooth size, arch size, and arch length have been considered. Other variables must be measured to accurately assess the true implications of arch dimensions on dental crowding. Additionally, the limited and inconsistent reporting of demographic variables such as age and ethnicity across the included studies restricted the ability to explore their potential influence on dental crowding and to perform subgroup analyses. The main limitation of this review was the difficulty in finding articles with comparable methods for data collection, measurement techniques, and data processing, which would allow for drawing accurate conclusions based on consistent results.

Additionally, the search strategy was limited to three electronic databases and restricted to studies published in English and Spanish within a defined time frame. Although these criteria were applied to ensure feasibility and focus on contemporary evidence, they may have led to the exclusion of relevant studies, including non-indexed or earlier foundational research, thereby increasing the risk of selection bias.

The inclusion of grey literature, such as academic theses, may introduce variability in methodological quality and reporting standards, potentially affecting reproducibility. However, these sources were included to reduce publication bias and to capture data from populations and contexts that are underrepresented in indexed journals. All included studies were subjected to the same eligibility criteria and risk-of-bias assessment, and the overall trends observed were consistent with those reported in peer-reviewed studies.

### Clinical implications

These findings may assist orthodontists in the diagnostic assessment of dental crowding by highlighting the importance of evaluating both tooth size and dental arch dimensions in an integrated manner. In particular, the lack of consistent sex-related differences suggests that clinical decisions should not rely solely on patient sex, but rather on individualized morphometric assessment. The evidence also highlights the relevance of considering age-related changes in arch dimensions when evaluating the progression of crowding.

The multifactorial nature of crowding may benefit from a preventive and interdisciplinary approach. Collaboration among orthodontists, pediatric dentists, periodontists, and even geneticists is essential to identify early indicators of developing malocclusion. Addressing growth patterns during mixed dentition, considering hereditary predispositions, and intervening before arch changes become irreversible can significantly reduce the need for future complex treatments.

Ultimately, a patient-centered approach that integrates dental and skeletal assessment with facial aesthetics and functional demands offers the best path toward achieving both effective treatment and long-term stability in managing dental crowding.

## Conclusions

Regarding the prevalence of crowding, most studies agree that there is no definitive consensus on whether it is more common in males or females. Nonetheless, the reviewed evidence confirms that dental arch dimensions are consistently associated with the presence and severity of crowding. Specifically, the combination of large tooth size and reduced arch dimensions is associated with a higher prevalence of dental crowding. Additionally, age-related arch length reduction is commonly associated with lower anterior crowding.

Therefore, tooth size, arch dimensions, and apical base length represent key factors to consider when planning individualized orthodontic treatment. However, the relative contribution and interaction of these variables remain insufficiently quantified due to the heterogeneity of the available evidence. Future research should prioritize longitudinal study designs with standardized measurement protocols and well-defined population characteristics in order to better determine the relative weight of each factor and to identify clinically relevant thresholds for predicting dental crowding.

## Author's note

This manuscript is part of the doctoral thesis currently being developed by Ana Isabel Contreras-Madrid as part of the Health Sciences Doctoral Program at the University of La Laguna.
