# Supplementary material for: Dental arch morphology and factors associated with dental crowding: a systematic review
Source: Front Oral Health. 2026 Jun 8;7:1784622. doi: 10.3389/froh.2026.1784622 (PMC13284102; doi:10.3389/froh.2026.1784622)
Supplement: Supplementary file 1 [file Table1.docx]

**Identification of studies via databases and registers**

Records removed *before screening*:

Duplicate records removed

(n = 113)

Records marked as ineligible by automation tools (n =0)

Records removed for other reasons (n =0)

Records identified from:

Databases PubMed, WOS, Scopus (n =978)

Registers (n =3)

Total: 981

**Identification**

Records screened by title

(n = 868)

Records excluded:

(n = 845)

Reports sought for retrieval

(n = 4)

Reports not retrieved

(n = 4)

**Screening**

Reports excluded (n = 9)

Reason 1 - omitting crowding or arch size variables (n = 2)

Reason 2 - analysing the accuracy and validity of the Bonwill-Hawley arch in crowding assessment (n = 2)

Reason 3 - exceeding the ten-year publication limit

(n = 1)

Reason 4 - addressing only prevalence (n = 2)

Reason 5 - dental crowding was not the primary outcome (n = 2)

Reports assessed for eligibility

(n = 23)

Studies included in review

(n = 14)

**Included**

Source: Page MJ, et al. BMJ 2021;372:n71. doi: 10.1136/bmj.n71.

This work is licensed under CC BY 4.0. To view a copy of this license, visit <https://creativecommons.org/licenses/by/4.0/>
